# Supplementary material for: Effects of Different Nutritional Patterns and Physical Activity on Body Composition: A Gender and Age Group Comparative Study
Source: Foods. 2024 Feb 8;13(4):529. doi: 10.3390/foods13040529 (PMC10888428; doi:10.3390/foods13040529)
Supplement: Supplementary file 1 [file foods-13-00529-s001.zip › foods-2832799-supplementary.pdf]

Table S1. Classification of Sports Activities by Category.

Endurance Sports	Skill Sports	Strength Training	Team Sports
Acquagym	Boxing	Calisthenics	Acrobatic Dance
Cycling	Capoeira	Functional Bodybuilding	Basketball
Elliptical Bike	Golf	Home Work Out	Handball
Hydrobike	Gymnastics	Pilates	Hockey
Rowing	Horse Riding	Powerlifting	Rugby
Running	Martial arts	Pump	Soccer
Spinning	Padel	Weightlifting	Volleyball
Step	Ping Pong		Waterpolo
Total Body	Pole Dance		
Treadmill	Skating		
Trekking	Tai Qui		
Walking	Tennis		
	Yoga		

Table S1. This table categorizes a diverse range of sports activities into four distinct groups: Endurance Sports, Skill Sports, Strength Training, and Team Sports. Each category is carefully curated to reflect the primary characteristics and demands of the sports listed, providing a clear and comprehensive overview of various physical activities.
